# Minocycline-induced microbiome alterations predict cafeteria diet-induced spatial recognition memory impairments in rats

**DOI:** 10.1038/s41398-020-0774-1

**Published:** 2020-03-13

**Authors:** Sarah-Jane Leigh, Nadeem O. Kaakoush, R. Frederick Westbrook, Margaret J. Morris

**Affiliations:** 1 School of Medical Sciences, UNSW Sydney, Sydney, NSW 2052 Australia; 2 School of Psychology, UNSW Sydney, Sydney, NSW 2052 Australia

**Keywords:** Hippocampus, Physiology, Diseases

## Abstract

Diets rich in sugar and saturated fat are associated with cognitive impairments in both humans and rodents with several potential mechanisms proposed. To test the involvement of diet-induced pro-inflammatory signaling, we exposed rats to a high-fat, high-sugar cafeteria diet, and administered the anti-inflammatory antibiotic minocycline. In the first experiment minocycline was coadministered across the diet, then in a second, independent cohort it was introduced following 4 weeks of cafeteria diet. Cafeteria diet impaired novel place recognition memory throughout the study. Minocycline not only prevented impairment in spatial recognition memory but also reversed impairment established in rats following 4 weeks cafeteria diet. Further, minocycline normalized diet-induced increases in hippocampal pro-inflammatory gene expression. No effects of minocycline were seen on adiposity or dietary intake across the experiments. Cafeteria diet and minocycline treatment significantly altered microbiome composition. The relative abundance of *Desulfovibrio*_OTU31, uniquely enriched in vehicle-treated cafeteria-fed rats, negatively and significantly correlated with spatial recognition memory. We developed a statistical model that accurately predicts spatial recognition memory based on *Desulfovibrio*_OTU31 relative abundance and fat mass. Thus, our results show that minocycline prevents and reverses a dietary-induced diet impairment in spatial recognition memory, and that spatial recognition performance is best predicted by changes in body composition and *Desulfovibrio*_OTU31, rather than changes in pro-inflammatory gene expression.

## Introduction

While it is known that excessive consumption of diets high in saturated fat and sugar is associated with cognitive impairment^1–3^ and increased risk of neurodegenerative disease^4,5^, the underlying causative mechanisms remain controversial. Substantial evidence indicates that such diets are associated with low-grade systemic^6,7^ and central^8^ inflammation, and diet-induced hippocampal pro-inflammatory changes are associated with impaired performance on hippocampal-dependent cognitive tasks in rats^9–13^ and mice^14–16^. However, other mechanisms, such as increased oxidative stress^13^, reduced sirtuin expression^12^ and neurotrophic factors^15,17,18^, and altered synaptic plasticity^19^ have also been implicated in diet-induced cognitive impairment. Therefore, further examination of the role of pro-inflammatory signaling in diet-induced cognitive impairment is warranted.

Minocycline, a bacteriostatic antibiotic and immunomodulator, readily crosses the blood brain barrier and inhibits microglial activation and proliferation^20^. Its administration effectively restores cognition in rodent models of chronic stress^21^, cranial irradiation^22^, aging^23^, and systemic bacterial infection^24^. Here, we sought to determine whether minocycline was also an effective treatment for spatial recognition memory impairments in rats fed a cafeteria diet. Two studies have previously examined the effect of minocycline on cognition in obese mice, using a passive avoidance^25^ and spatial recognition and navigation tasks^26^. The first task confounds hippocampal contextual learning with locomotor and anxiety-like behavior, the latter of which was shown to be reduced in an elevated plus maze task in these same mice^25^, and both studies administered minocycline for no more than 2 weeks^25,26^. It is unknown whether minocycline can prevent diet-induced cognitive impairment in rodents prior to induction of obesity or reverse defects once these arise, and whether these drug-induced improvements are long-lasting.

In addition, minocycline has antibiotic activity and has been recently reported to reduce some effects of high-fat diet in rats through altering food intake and the gut microbiome composition^27^. Given the role of the gut microbiome in diet-induced cognitive impairment^11,28,29^, it is important to examine the effects of minocycline on microbiome composition and to investigate potential behavioral associations as these have not been previously studied.

First, we aimed to determine whether chronic oral minocycline treatment would protect rats from developing diet-induced cognitive impairment (experiment 1), as well as reverse already established cafeteria diet-induced cognitive impairment (experiment 2). Secondly, we investigated potential mechanisms by which minocycline may be modulating cognition. Specifically, we examined associations between spatial recognition memory and metabolic impairments, white adipose (WAT) and hippocampal pro-inflammatory signaling gene expression, and changes to fecal microbiome composition. Finally, we used predictive modeling to identify key predictors of spatial recognition memory.

## Methods

### Ethics statement

The experimental protocol was approved by the Animal Care and Ethics Committee of the University of New South Wales in accordance with the guidelines for the use and care of animals for scientific purposes (Australian National Health and Medical Research Council).

### Subjects and experimental manipulations

One hundred and eight male Sprague–Dawley rats aged 8 weeks (190–220 g; Animal Resource Centre, Australia) were housed at 18–22 °C (12 h light/dark cycle) and maintained *ad libitum* on water and standard chow (11 kJ/g; 65% carbohydrate, 22% protein and 13% fat; Premium Rat Maintenance diet, Gordon’s Stockfeeds, Australia).

Following acclimatization, weight-matched groups were randomly allocated to treatments. Diets comprised standard chow or cafeteria diet (full protocol in ref. ^30^,) consisting of chow and water, 10% sucrose solution and a selection of cakes, biscuits and protein sources (e.g., meat pie, dim sims, and dog food) that varied daily.

Body weight and food intake were measured twice weekly. Body composition was assessed by EchoMRI-900 (EchoMRI LLC, USA). For intake measures, foods were weighed before presentation, and reweighed 24 h later. Energy intake was calculated from manufacturers’ information assuming equal intake per cage.

Minocycline hydrochloride (Chemlin, China; 40 mg/kg/day) was delivered via daily syringe feeding^31,32^. Minocycline was suspended in vehicle (golden syrup; CSR Sugar, Australia; diluted to 20–35% sucrose solution with water) immediately before administration.

**Fig. 1:**
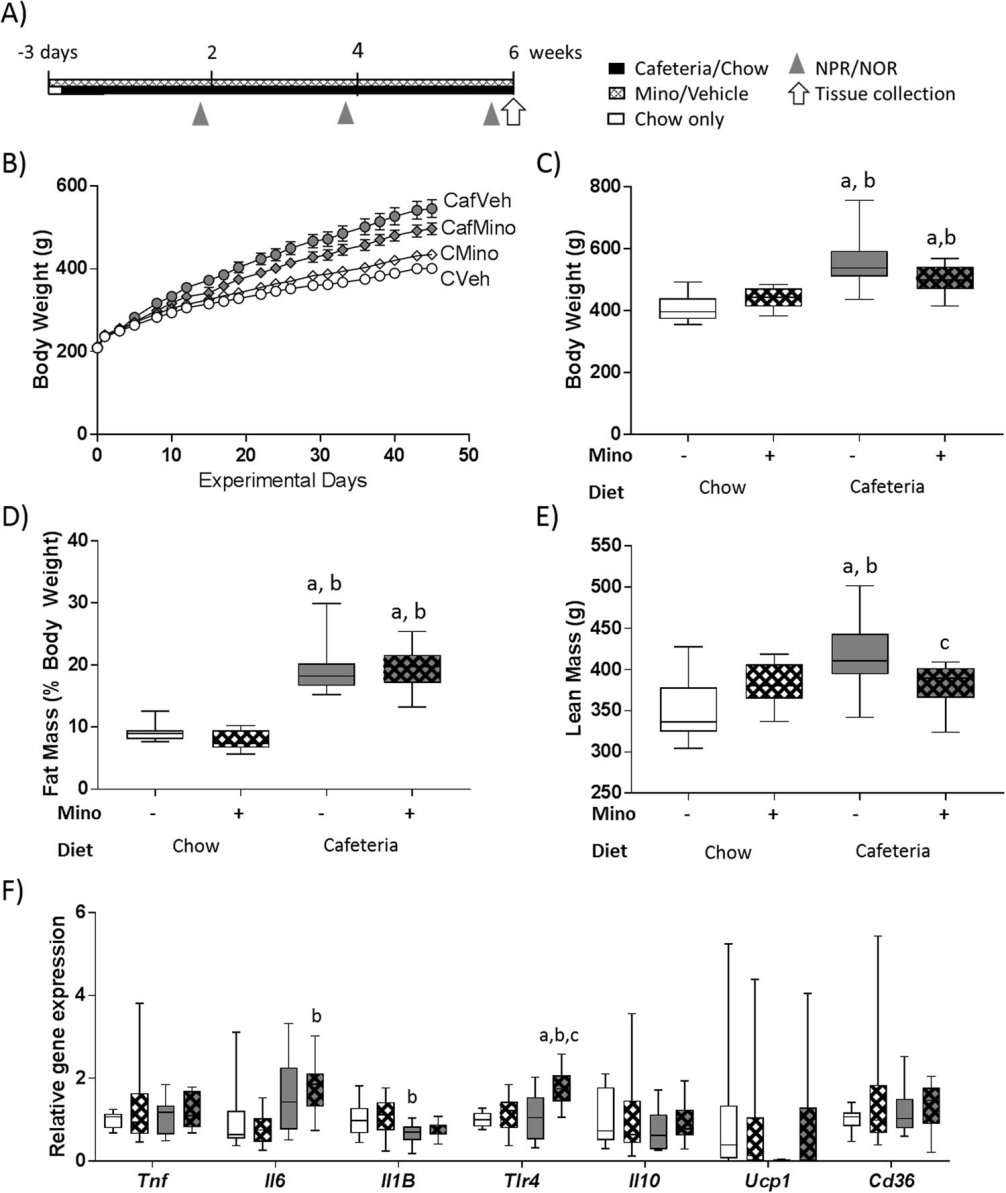
Minocycline treatment does not affect adiposity or white adipose pro-inflammatory gene expression in cafeteria-fed rats. **a** Timeline for study. **b** Body weight over the experiment, and **c** body weight, **d** fat mass (as a percentage of body weight), and **e** absolute lean mass following 6 weeks of diet exposure. **f** Pro- and anti-inflammatory and uncoupling protein 1 gene expression in retroperitoneal white adipose tissue. Cd36 cluster of differentiation 36, Il6 interleukin-6, Il1B interleukin-Iβ, Il10 interleukin-10, Tlr4 toll-like receptor 4, Ucp1 uncoupling protein 1. Data expressed as box-and-whisker plots (min, IQR, max); *n* = 11–12; data were analyzed by two-way ANOVA followed by Tukey-adjusted post hoc testing. ^a^*p* < 0.05 relative to CVeh, ^b^*p* < 0.05 relative to CMino, ^c^*p* < 0.05 relative to CafVeh.

Experiment 1 (Fig. 1a) examined the effects of minocycline administration during cafeteria diet exposure on spatial recognition impairment. Following a 2 × 2 design (*n* = 12 rats/group), rats were exposed to vehicle (Veh) or minocycline (Mino) for 3 days (to monitor for antibiotic-induced gastrointestinal symptoms) while consuming standard chow (C) before placing half the rats onto cafeteria diet (Caf) for 6 weeks while maintaining drug treatment.

Experiment 2 followed a 2 × 2 design (*n* = 12 rats/group) and investigated the effect of minocycline administration on rats with an established diet-induced spatial recognition impairment. Rats were assigned to C or Caf for 4 weeks, before half were placed on either Veh or Mino for an additional 4 weeks while maintaining their diet. A fifth group (*n* = 12, CafMino*) was placed onto both cafeteria diet and minocycline for the final 4 weeks, to replicate the same conditions of Experiment 1.

### Behavior—novel object and novel place tasks

Hippocampal-dependent spatial and perirhinal-dependent object recognition memory were assessed in a square arena (60 cm × 60 cm × 60 cm; 40 lux) using novel place (NPR) and novel object (NOR) tasks, respectively^33,34^. Previously, we showed that cafeteria-fed rats exhibit impaired NPR but intact NOR^12,13^. For the NOR, objects were matched on volume and color, and differed in shape and material. Object type and locations were counterbalanced across treatment groups and across time, and videos were relabeled to blind the experimenter prior to scoring. For 2 days prior to the first task rats were exposed to the empty arena for 10 min each day.

Both NOR and NPR tasks consisted of familiarization, retention and test phases. In the familiarization phase, rats were placed into the arena with two identical novel objects and allowed to explore for 5 min. They returned to their home cage for a 5-min retention period, then the 3-min test began. For the NOR, objects were placed in the same locations as familiarization but one object was novel while the other was identical to that used during familiarization. In the NPR, the objects were identical to those shown during familiarization but one object was placed in a novel location, the other remained in its original position. Exploration ratio was calculated for each rat as (novel exploration time)/(novel + familiar exploration time). Following testing, fecal samples were collected either from the arena or the rectum.

### Sample collection

Rats were deeply anaesthetized (ketamine/xylazine 15/100 mg/kg intraperitoneally) and body weight, naso-anal length, girth, and blood glucose measured. Cardiac puncture was performed and rats were decapitated. The dorsal hippocampus was dissected (within a coronal block defined by rostro-caudal limits of the Circle of Willis). Liver, retroperitoneal and gonadal WAT were dissected and weighed. Feces were collected from the distal descending colon. Samples and plasma were snap-frozen in liquid nitrogen and stored at −80 °C.

### Plasma hormone, triglyceride and high-density lipoprotein measurements

Plasma leptin, insulin, and high-density lipoprotein cholesterol concentrations were determined according to manufacturer’s instructions (CrystalChem Inc., USA). Plasma triglyceride concentration was determined using triglyceride reagent (Roche Diagnostics, Australia) against glycerol standard (Sigma-Aldrich Pty Ltd., Australia).

### RNA extraction and gene-expression assays

RNA was extracted from dorsal hippocampus and retroperitoneal WAT using TRI Reagent (Sigma-Aldrich Pty Ltd., Australia). Following DNAse I treatment (Merck, Australia) 1.5 or 2 µg of RNA (WAT and hippocampus, respectively) were reverse transcribed to cDNA (Thermofisher Scientific, USA). Gene expression was assessed using TaqMan inventoried gene expression assays (Life Technologies Australia Pty Ltd, Australia). Genes of interest (full list in Supplementary Table 1) were normalized using the geometric mean of the most stable housekeeping genes (*Ywhaz* and *Hprt1* for hippocampus, *Gapdh* and *Hprt1* for WAT) identified by Normfinder^35^. Analysis of relative gene expression was performed using the ∆∆C_T_ method normalized to an independent calibrator ^36^.

### Statistical analyses

Results were analyzed using Welch-corrected *t* tests or two-way between-subjects ANOVA, while measures over time were analyzed using three-way mixed ANOVA. Post hoc pairwise comparisons were performed using a Tukey adjustment where appropriate (THSD). Where data violated homoscedasticity of variance, log transformations were employed. Pearson correlations assessed the relationship between place task performance and measures of interest in the study. All analyses were completed using IBM SPSS Statistics 23 (Australia).

### Fecal DNA extraction, microbiome community sequencing, and statistical analyses

DNA extraction was performed using the PowerFecal DNA Isolation Kit (MoBio Laboratories, Carlsbad, CA, USA) and composition of the microbial communities was assessed by Illumina amplicon sequencing (2 × 250 bp MiSeq chemistry, V4 region, 515F-806R primer pair) using a standard protocol^37^. The sequence data were then analyzed using MOTHUR^38^ based on modified commands from MiSeq SOP^39^. Sequence data were subsamples to *n* = 10,038 total clean reads/sample.

Operational taxonomic unit (OTU) correlations and LEfSe analyses were completed using Calypso^40^, where multiple testing was corrected using the Benjamini Hochberg false-discovery rate procedure (FDR)^41^. Alpha diversity metrics were obtained from Calypso and analyses were completed using IBM SPSS Statistics, while nonmetric multidimensional scaling and DESeq2 were performed using R. The R package Phyloseq^42^ was used for the negative binomial Walk test in DESeq2^43^ with an FDR correction applied.

Distanced-based linear modeling, nonmetric multidimensional scaling and PERMANOVA were completed using Primer (version 6; Primer-E Ltd., UK^44^; all using a Bray–Curtis similarity matrix at the OTU level). Marginal and sequential distance-based linear modeling interrogate the unique and conditional contributions of each predictor variable to the variance in the Bray–Curtis similarity matrix.

OTU abundances were analyzed using SPSS with Kruskal–Wallis tests when necessary, followed by nonparametric Bonferroni–Dunn post hoc testing. OTUs of interest were identified using SINA Aligner ^45^.

### Predictive modeling

NPR performance was predicted from models based on data from Experiment 1 along with CafMino* yielding 112 observations from *N* = 60 rats which were combined into a training dataset comprising NPR, NOR, body composition, OTUs associated with NPR performance, and bacterial species richness and evenness. Multiple regression models, random forest algorithms^46^ and gradient boosting algorithms^47^ were trialed in R, and the best model (multiple regression: Place task performance ~ Fat mass + *Desulfovibrio*_OTU31) was selected. This model was then assessed on the test dataset, comprising all observations from Experiment 2 except the CafMino* group (89 observations from *N* = 48 rats).

## Results

### Minocycline reduced lean mass without changing energy intake, adiposity or white adipose gene expression in cafeteria-fed rats (Experiment 1)

Terminal body weight (Fig. 1c) was significantly elevated in CafVeh rats compared to chow-fed groups (both *p*’s < 0.001) as well as CafMino rats compared to CVeh (*p* < 0.001) and CMino (*p* = 0.036: THSD; DietXDrug: *F*_1,43_ = 7.116, *p* = 0.011). Cafeteria diet exposure significantly elevated total energy, carbohydrate and fat intake irrespective of minocycline treatment with no group differences in absolute protein intake (Table 1). All rats gained weight over the 6-week study (Fig. 1b).

**Table 1:** Energy intake, anthropometric measures at tissue collection and plasma measures.

Measure	Chow diet		Cafeteria diet		*p* Values		
	Vehicle	Minocycline	Vehicle	Minocycline	Diet	Mino	Interaction
*Total energy and macronutrient intake (average kJ/rat)*							
Total energy intake	14432 ± 282	15292 ± 989	45584 ± 2628^a,b^	41886 ± 719^a,b^	<0.001	–	–
Total protein intake	3140 ± 67	3269 ± 211	3768 ± 150^a^	3355 ± 64	0.023	–	–
Total carbohydrate intake	9419 ± 200	9806 ± 632	25292 ± 1407^a,b^	23574 ± 334^a,b^	<0.001	–	–
Total fat intake	1952 ± 41	2032 ± 131	13400 ± 719^a,b^	11998 ± 373^a,b^	<0.001	–	–
*Anthropometric measures (cm)*							
Naso-anal length	23.8 ± 0.3	24.6 ± 0.3	25.2 ± 0.2^a^	24.9 ± 0.2^a^	0.002	–	0.034
Girth	17.7 ± 0.2	18.1 ± 0.1	20.5 ± 0.3^a^	19.9 ± 0.4^a^	<0.001	–	–
Tibia length	4.10 ± 0.03	4.10 ± 0.06	4.17 ± 0.04	4.12 ± 0.04	–	–	–
Organ weights (g)							
Liver weight	14.3 ± 0.4	15.4 ± 0.6	22.3 ± 1.4^a,b^	23.0 ± 1.4^a,b^	<0.001	–	–
Heart weight	1.04 ± 0.04	1.12 ± 0.03	1.38 ± 0.04^a,b^	1.29 ± 0.11^a,b^	<0.001	–	0.035
Fat pad weights (g)							
Retroperitoneal	4.03 ± 0.26	3.48 ± 0.23	14.67 ± 1.53^a,b^	12.66 ± 1.15^a,b^	<0.001	–	–
Gonadal	4.23 ± 0.35	3.94 ± 0.27	14.62 ± 1.47^a,b^	12.04 ± 1.31^a,b^	<0.001	–	–
Total	8.27 ± 0.55	7.43 ± 0.47	29.28 ± 2.86^a,b^	24.70 ± 2.26^a,b^	<0.001	–	–
*Unfasted blood and plasma measures*							
Blood glucose (mmol/L)	9.5 ± 0.4	9.8 ± 0.4	10.2 ± 0.4	10.6 ± 0.4	–	–	–
Plasma insulin (ng/mL)	1.55 ± 0.15	1.16 ± 0.05	2.62 ± 0.43^a,b^	1.85 ± 0.21	0.002	0.034	–
Plasma leptin (ng/mL)	3.65 ± 0.30	3.35 ± 0.28	10.65 ± 1.75^a,b^	12.39 ± 1.44^a,b^	<0.001	–	–
Plasma triglyceride (mmol/L)	0.86 ± 0.09	0.75 ± 0.07	2.84 ± 0.28^a,b^	2.14 ± 0.22^a,b^	0.034	<0.001	–
Plasma high-density lipoprotein (mmol/L)	0.56 ± 0.03	0.46 ± 0.03	0.45 ± 0.04	0.50 ± 0.04	–	–	0.026

Data expressed as mean ± SEM; *n* = 4 cages for energy intake measures; *n* = 10–12 for other measures. Data were analyzed using two-way ANOVA, followed by post hoc multiple comparisons with a Tukey HSD correction.

^a^*p* < 0.05 relative to CVeh.

^b^*p* < 0.05 relative to CMino.

^c^*p* < 0.05 relative to CafVeh.

Relative fat mass (Fig. 1d) was significantly elevated in cafeteria-fed groups versus chow-fed groups (all THSD *p*’s < 0.001; diet: *F*_1,43_ = 184.969, *p* < 0.001) and cafeteria diet significantly increased girth, liver weight, and all fat depot weights examined irrespective of drug treatment (Table 1). In contrast, cafeteria diet-induced increases in plasma insulin and triglyceride concentrations were reduced by minocycline (Table 1). Absolute lean mass was significantly increased in CafVeh rats relative to all other groups (diet × drug: *F*_1,43_ = 9.141, *p* = 0.004; Fig. 1e), implying that minocycline treatment restricted growth in cafeteria-fed rats.

Retroperitoneal WAT inflammatory signaling and browning genes were assessed to determine whether minocycline regulates pro-inflammatory signaling in WAT (Fig. 1f), as these changes are known to contribute to diet-induced cognitive impairment^48^. WAT *Tlr4* expression was uniquely upregulated in CafMino rats relative to all other groups (CVeh: *p* < 0.001, CMino: *p* = 0.004, CafVeh: *p* = 0.002 (THSD); DietXDrug: *F*_1,42_ = 5.025, *p* = 0.030). In contrast, expression of both *Il6* and *Il1B* were significantly changed by cafeteria diet overall independently of minocycline treatment: *Il6* was significantly increased by cafeteria diet (*F*_1,42_ = 12.118, *p* = 0.001) while *Il1B* expression was reduced by that diet (*F*_1,42_ = 7.877, *p* = 0.008). No significant differences were observed in *Tnf*, *Il10*, *Ucp1*, and *Cd36* expression.

### Minocycline prevented cafeteria diet-induced spatial recognition impairments and altered hippocampal pro-inflammatory gene expression

**Fig. 2:**
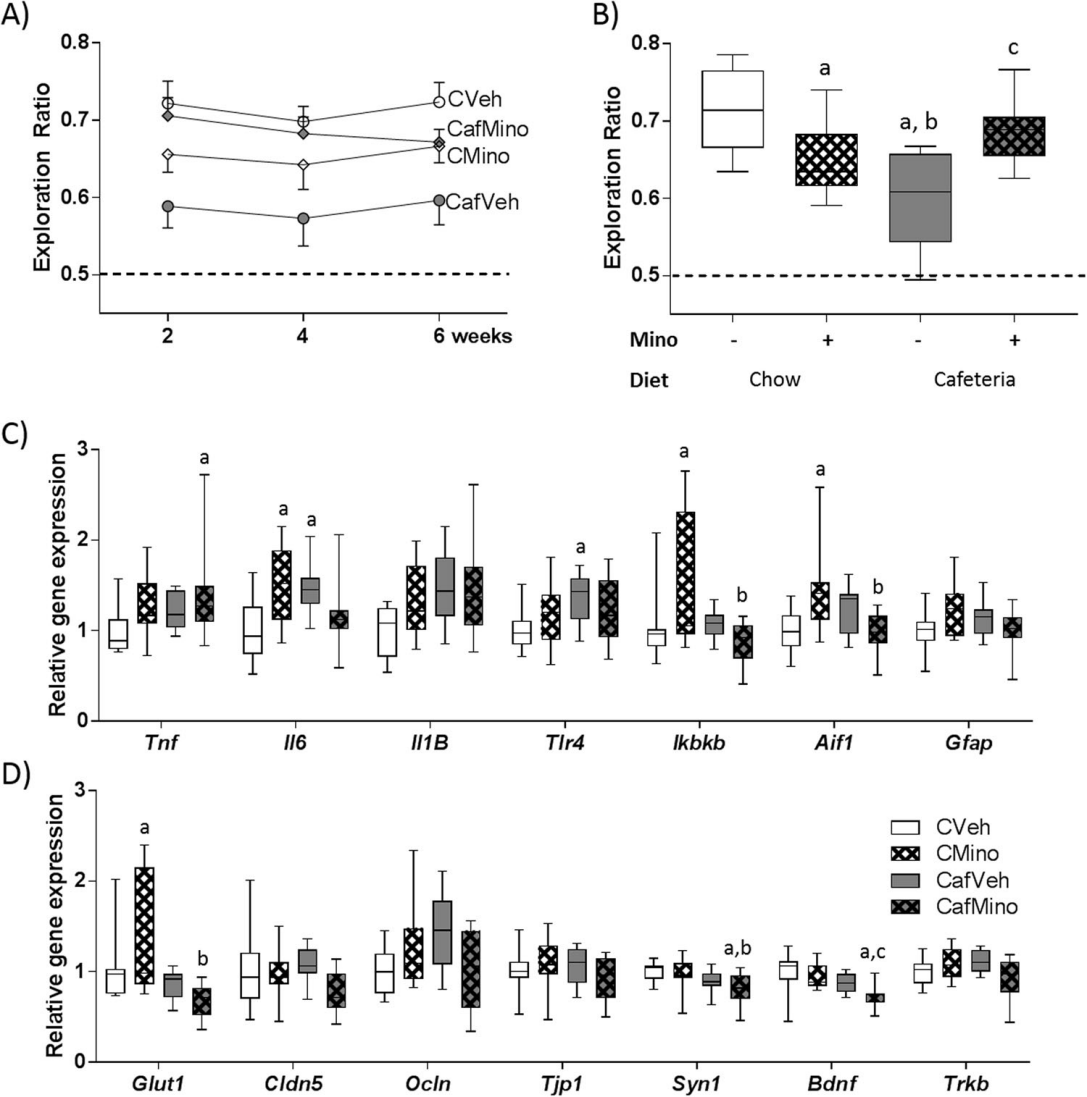
Minocycline treatment improves spatial recognition memory and hippocampal pro-inflammatory gene expression in cafeteria-fed rats. **a** Novel place task performance over the study and **b** average place task performance expressed as exploration ratios. Relative gene expression of **c** pro-inflammatory signals and **d** markers for blood brain barrier integrity, synaptic plasticity and neurogenesis in the dorsal hippocampus. Aif1 allograft inflammatory factor 1, Bdnf brain derived neurotrophic factor, Cldn5 claudin 5, Gfap glial fibrillary acidic protein, Glut1 glucose transporter 1, Ikbkb inhibitor of nuclear factor kappa B kinase subunit beta, Il6 interleukin-6, Il1B interleukin-Iβ, Ocln occludin, Syn1 synapsin 1, Tlr4 toll-like receptor 4, Tjp1 tight junction protein 1, Tnf tumor necrosis factor a, Trkb tropomyosin receptor kinase. Data expressed as box-and-whisker plots (min, IQR, max); *n* = 11–12; data were analyzed by two-way ANOVA followed by Tukey-adjusted post hoc testing. ^a^*p* < 0.05 relative to CVeh, ^b^*p* < 0.05 relative to CMino, ^c^*p* < 0.05 relative to CafVeh.

NPR and NOR were assessed at 2, 4, and 6 weeks of cafeteria diet exposure (Fig. 1a). Minocycline treatment reduced NPR performance in chow-fed rats while improving performance in cafeteria-fed rats (diet × drug: *F*_1,80_ = 17.703, *p* < 0.001) with no significant effect of time (*F*_2,80_ < 1; Fig. 2a). When NOR was examined, there were no significant effects of diet, minocycline treatment, or time (Supplementary Fig. 1B). Since there were no effects of time on either NPR or NOR measures, tests were averaged to reduce variability for further analyses (Fig. 2b, Supplementary Fig. 1A). No significant group effects were observed for total test exploration times on either tasks (Supplementary Fig. 1C, D).

Pro-inflammatory gene expression was assessed in the hippocampus (Fig. 2c): *Il6*, *Ikbkb*, *Aif1* (marker of microglial proliferation^49^) and *Gfap* (marker of astrocyte proliferation and central nervous system injury^50^) followed a similar pattern where minocycline reduced gene expression in cafeteria-fed rats, but increased that expression in chow-fed rats (DietXDrug: *F*_1,42_ = 14.56, *p* < 0.001; *F*_1,41_ = 7.204, *p* = 0.01; *F*_1,41_ = 13.592, *p* = 0.001 and *F*_1,41_ = 6.395, *p* = 0.015, respectively). In contrast, *Il1B* and *Tlr4* gene expression were significantly upregulated by cafeteria diet (diet: *F*_1,42_ = 6.397, *p* = 0.015 and *F*_1,42_ = 4.557, *p* = 0.039, respectively) with no minocycline-induced differences. Relative *Tnf* expression was significantly elevated by minocycline overall (*F*_1,42_ = 4.585, *p* = 0.038), and CafMino rats exhibited significantly higher *Tnf* expression relative to CVeh rats (*p* = 0.043; THSD).

Expression of markers of blood brain barrier integrity and glucose transport were assessed (Fig. 2d), as emerging evidence implicates these processes in diet-induced cognitive impairment^51,52^. This was not supported by our data, which showed minocycline-induced, but not cafeteria diet-induced, changes in gene expression. *Ocln* expression exhibited an interaction between minocycline and cafeteria diet (DietXDrug: *F*_1,42_ = 8.094, *p* = 0.007), while *Cldn5* expression was reduced by minocycline treatment overall (*F*_1,42_ = 4.248, *p* = 0.046). *Glut1* expression was upregulated by minocycline in chow-fed rats relative to CafVeh (*p* = 0.017) and CafMino (*p* < 0.001, THSD; DietXDrug: *F*_1,41_ = 6.135, *p* = 0.017). No significant group differences were observed for *Tjp1* expression. *Syn1* expression was significantly reduced by cafeteria diet overall (*F*_1,42_ = 9.988, *p* = 0.003) with no effects of minocycline, while *Bdnf* expression was significantly reduced by both cafeteria diet exposure (*F*_1,40_ = 12.349, *p* = 0.001) and minocycline treatment (*F*_1,40_ = 5.226, *p* = 0.028) independently. No significant differences were observed in *Trkb* expression.

When correlations between average NPR performance and other study variables were assessed, several relationships were observed (Supplementary Table 2). Overall, measures of increased adiposity were negatively correlated with NPR performance, while WAT *Ucp1* gene expression was positively associated with NPR performance. When all vehicle-treated rats were considered, negative associations between NPR performance and measures of adiposity, and hippocampal *Il6* and *Il1B* expression were observed.

### Cafeteria diet and minocycline treatment both impacted microbial species diversity and microbiome composition

**Fig. 3:**
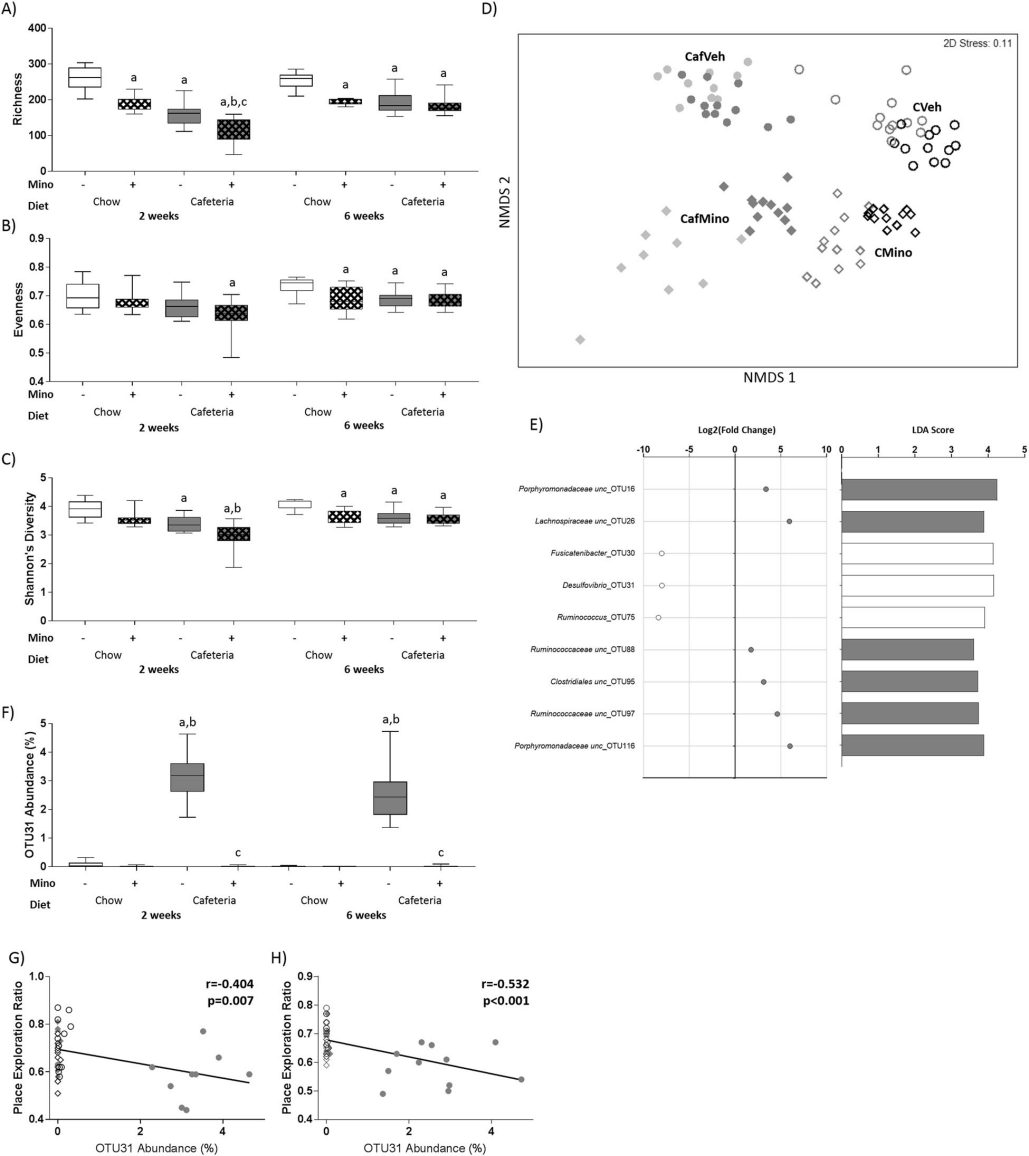
Impact of cafeteria diet and minocycline on fecal microbiota at 6 weeks. **a** Microbial species richness, **b** evenness, and **c** Shannon’s diversity at 2 and 6 weeks. Data expressed as box-and-whisker plots (min, IQR, max); *n* = 11–12 for individual measures; data were analyzed by three-way ANOVA followed by Tukey-adjusted post hoc testing. **d** Nonmetric multidimensional scaling (Bray–Curtis, 1000 permutations) showing similarity between fecal microbiota samples at 2 (light gray) and 6 (dark gray/black) weeks. **e** Selected OTUs identified by both DESeq2 (adjusted *p* < 0.05) and LEfSe (LDA score > 2, *p* < 0.05) as differentially abundant with minocycline in cafeteria-fed rats that are significantly correlated with place task performance. The color of the circle/bar denotes the group the OTU is enriched in (open: vehicle; gray: minocycline). Complete list in Supplementary Table 2. **f** Relative abundance of OTU31 at 2 and 6 weeks. Data expressed as box-and-whisker plots (min, IQR, max); *n* = 11–12 for individual measures; data were analyzed using Kruskal–Wallis test followed by nonparametric Dunn–Bonferroni post hoc testing. Scatterplots for place task performance and relative abundance of OTU31 at (**g**) 2 and (**h**) 6 weeks, showing overall lines of best fit; *n* = 46. Post hoc symbols: ^a^*p* < 0.05 relative to CVeh, ^b^*p* < 0.05 relative to CMino, ^c^*p* < 0.05 relative to CafVeh.

Fecal microbiome analysis was conducted at 2 and 6 weeks of diet exposure. Measures of microbial α-diversity across time were assessed using a three-way ANOVA, including richness, evenness and Shannon’s Diversity (Fig. 3). Microbial species richness (Fig. 3a) exhibited significant interactions between cafeteria diet exposure and minocycline treatment (*F*_1,39_ = 8.665, *p* = 0.005), time and diet (*F*_1,39_ = 22.647, *p* < 0.001), and time and drug treatment (*F*_1,39_ = 5.261, *p* = 0.027) where both minocycline and cafeteria diet reduced richness, but these effects were less severe over time. Evenness (Fig. 3b) significantly increased over time (*F*_1,39_ = 11.613, *p* = 0.002), and was significantly reduced by cafeteria diet (*F*_1,39_ = 12.218, *p* = 0.001) and minocycline treatment (*F*_1,39_ = 8.918, *p* = 0.005) overall. Shannon’s diversity (Fig. 3c) was significantly reduced by diet (*F*_1,39_ = 40.101, *p* < 0.001) and minocycline treatment (*F*_1,39_ = 24.979, *p* < 0.001) overall, and a significant interaction between time and diet was observed (*F*_1,39_ = 6.957, *p* = 0.012).

Microbiome composition at the OTU level was significantly affected by cage (within diet; Pseudo-*F*_3,59_ = 1.479, *p* = 0.001) and time (Pseudo-*F*_1,59_ = 7.109, *p* = 0.001) effects as well as a significant interaction between cafeteria diet and minocycline treatment (Pseudo-*F*_1,59_ = 9.093, *p* = 0.001) when assessed using 4-way PERMANOVA (999 permutations). Nonmetric multidimensional scaling and pairwise comparisons confirmed that the microbiome compositions of all groups significantly differed from each other (all *p*’s<0.001; Fig. 3d).

When variables were assessed for their unique contribution to the variance in overall microbiome composition, several adiposity measures, as well as retroperitoneal *Il6* and *Tlr4* gene expression, hippocampal *Il1B* expression, and NPR performance were identified as significant predictors of global microbiome composition (Supplementary Table 3A). When the conditional contribution of variables of interest to overall microbiome composition was assessed while controlling for diet and minocycline exposure, in addition to several significant adiposity measures, both hippocampal *Il6* expression (*R*^2^ = 0.029, p = 0.032) and NPR performance (*R*^2^ = 0.026, *p* = 0.007) were significant predictors of microbiome composition at the OTU level (Supplementary Table 3B, complete model predicts 47.6% of the variance in microbiome composition).

DESeq2 and LEfSe analyses were used to identify differentially enriched OTUs with diet exposure amongst the top 200 OTUs at week 6. In cafeteria-fed rats, minocycline treatment depleted several OTUs from *Lactobacillus* as well as *Blautia* genera, and enriched several OTUs from *Lachnospiraceae* and *Porphyromonadaceae* families (Supplementary Table 4). The significantly enriched and depleted OTUs that are also significantly associated with NPR performance are shown in Fig. 3e.

*Desulfovibrio*_OTU31 was selected for further investigation as this OTU was strongly associated with NPR performance at 2 (*r* = −0.404, *p* = 0.007, Fig. 3g) and 6 (*r* = −0.532, *p* < 0.001, Fig. 3h) weeks of cafeteria diet. *Desulfovibrio*_OTU31 was uniquely enriched in CafVeh rats across the study (Fig. 3f). SINA aligner analysis showed that *Desulfovibrio*_OTU31 shared 99.8% sequence identity with *Desulfovibrio piger* strain ATCC 29098.

### Minocycline effects on established cafeteria diet-induced cognitive impairment (Experiment 2)

**Fig. 4:**
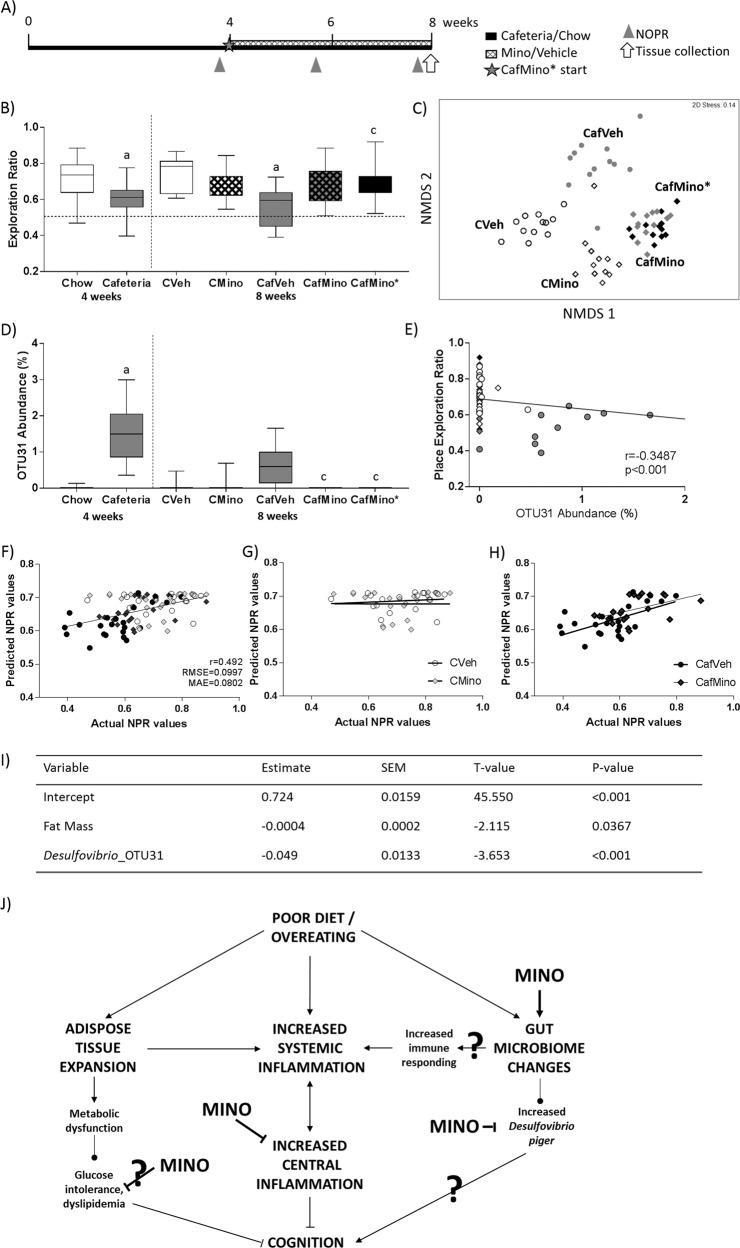
Impact of minocycline treatment following 4 weeks of cafeteria diet on place task performance, fat mass and microbiota. **a** Study timeline. **b** Average novel place task performance post-minocycline treatment expressed as exploration ratios. Data expressed as box-and-whisker plots (min, IQR, max); *n* = 11–12; data were analyzed by two-way ANOVA followed by Tukey-adjusted post hoc testing. ^a^*p* < 0.05 relative to CVeh, ^b^*p* < 0.05 relative to CMino, ^c^*p* < 0.05 relative to CafVeh. **c** Nonmetric multidimensional scaling (Bray–Curtis, 1000 permutations) showing similarity between fecal microbiota samples at 8 weeks. **d** Relative abundance of OTU31 at 4 and 8 weeks. Data expressed as box-and-whisker plots (min, IQR, max); analyzed using Kruskal–Wallis test followed by nonparametric Dunn–Bonferroni post hoc testing. ^a^*p* < 0.05 relative to Chow at 4 weeks, ^c^*p* < 0.05 relative to CafVeh at 8 weeks. **e** Scatterplot of Pearson correlation between place exploration ratio and relative OTU31 abundance, showing overall line of best fit; *n* = 10–12. o Vehicle-treated animals (open: chow, gray: cafeteria); ◊: minocycline treated animals (open: chow, gray: cafeteria, black: CafMino*). **f** Prediction of place task performance using final multiple regression model overall and for **g** chow-fed and **h** cafeteria-fed rats. **i** Final multiple regression model based on body composition and OTUs of interest. Training dataset: 112 observations from *N* = 60 rats. Test dataset: *n* = 89 observations from *N* = 48 rats. r correlation coefficient, RMSE root mean square error, MAE mean absolute error. CafMino* represents a replication group for the previous study, where rats were placed on both cafeteria diet and minocycline when all other rats were allocated to vehicle or minocycline. **j** Poor diet and overeating lead to increased adiposity and systemic low-grade inflammation, as well as altered gut microbiota. Systemic inflammation is highly interrelated with central inflammatory tone, which was inhibited by minocycline treatment. Changes to the gut microbiome include altered composition, which may contribute to the diet-induced state of systemic inflammation. Cafeteria diet enriched *Desulfovibrio piger* which was depleted by minocycline and negatively associated with place task performance in both experiments. Minocycline treatment improved short-term spatial recognition memory, measured by the novel place recognition task. Pointed arrow: promotion of the downstream process; blunted arrow: inhibition of the downstream process.?: hypothesized mechanism.

Experiment 2 examined whether minocycline could reverse already established diet-induced impairment in the NPR task (Fig. 4a). Rats were fed chow or cafeteria diet for 4 weeks, resulting in a doubling of relative fat mass compared to chow (chow: 8.22 ± 0.36%, cafeteria: 18.01 ± 1.06%; *t*_28.39_ = 7.75, *p* < 0.001). While maintaining their diet, rats were treated daily with either vehicle or minocycline for a further 4 weeks.

Four weeks of cafeteria diet significantly impaired NPR performance relative to chow-fed controls (*t*_55.26_ = 4.435, *p* < 0.001; Fig. 4b) while minocycline significantly improved average NPR in cafeteria-fed (diet × drug: *F*_1,40_ = 12.799, *p* = 0.001) but did not affect chow-fed rats. The energy intake, metabolic and adiposity measures are shown in Supplementary Table 5.

Microbiome composition at the end of Experiment 2 was significantly altered by cafeteria diet (Pseudo-*F*_1,44_ = 7.718, *p* = 0.026), minocycline (Pseudo-*F*_1,44_ = 7.558, *p* = 0.031), and cage (within diet; Pseudo-*F*_3,44_ = 1.472, *p* = 0.002) when 3-way PERMANOVA and non-metric multidimensional scaling (999 permutations; Fig. 4c). FDR-corrected pairwise comparisons showed that all treatment groups were significantly different (all *p*’s = 0.001) except for CafMino and CafMino* groups, which did not differ significantly in microbiome composition (*p* = 0.222).

Associations between OTU abundances, NPR performance and adiposity measures were investigated to determine whether specific OTUs consistently predicted group differences in these variables over the experiment. Two OTUs were significantly correlated with NPR overall, *Phascolarctobacterium*_OTU7 and *Desulfovibrio*_OTU31 (the latter also seen in Experiment 1, Fig. 3g, h). Follow-up comparisons showed that *Desulfovibrio*_OTU31 was enriched in cafeteria-fed rats prior to minocycline exposure (Cafeteria in Fig. 4d), and in CafVeh rats following 4 weeks’ minocycline treatment (Fig. 4d). Further, its relative abundance was negatively associated with NPR performance overall (*r* = −0.324, *p* < 0.001; Fig. 4e), as observed in Experiment 1.

### Fat mass and Desulfovibrio_OTU31 predict place task performance across experiments

Given the associational relationships between specific OTU abundances, adiposity and NPR, we then used multiple regression and machine learning algorithms to predict NPR performance in Experiment 2, using the results from rats treated with minocycline alongside diet exposure (Experiment 1 and CafMino*). Using NPR performance to calculate baseline error in prediction (estimate = 0.675, root mean square error (RMSE) = 0.1146, mean absolute error (MAE) = 0.0935), we found that a reduced multiple regression model (Fig. 4i; *R*^2^ = 0.185) predicted NPR performance for the training dataset (Experiment 1 and CafMino*) above chance. This model also performed best when predicting NPR performance in the test dataset (Experiment 2; *R*^2^ = 0.242; RMSE = 0.100, MAE = 0.080; Fig. 4f), although the model best predicted NPR performance in the cafeteria-fed rats (Fig. 4h; CafVeh: *r* = 0.596, p = 0.003; CafMino: *r* = 0.473, *p* = 0.026) with limited predictive value in chow-fed groups (Fig. 4g; CVeh: *r* = 0.136, *p* = 0.567; CMino: *r* = −0.002, *p* = 0.991). The reduced multiple regression model identified absolute fat mass and *Desulfovibrio*_OTU31 as significant predictors of spatial recognition memory. Figure 4j features a schematic of the mechanisms underlying diet-induced cognitive dysfunction and the observed actions of minocycline.

## Discussion

In Experiment 1, minocycline protected NPR performance in cafeteria-fed rats when administered prophylactically over 6 weeks, without affecting energy intake, relative adiposity or NOR performance. Several pro-inflammatory genes were downregulated in the hippocampus by minocycline in cafeteria-fed rats and NPR performance was negatively associated with *Il1b* and *Il6* gene expression. Both minocycline and cafeteria diet altered bacterial species diversity and microbiome composition, and the relative abundance of *Desulfovibrio*_OTU31 was negatively associated with NPR performance. In Experiment 2, established cafeteria diet-induced spatial recognition memory impairments were reversed with minocycline, and minocycline exerted similar effects on the microbiome in cafeteria-fed rats irrespective of whether drug treatment was started prior to (CafMino*) or following cafeteria diet exposure (CafMino).

Predictive modeling identified *Desulfovibrio*_OTU31 and fat mass as key factors predicting NPR performance in cafeteria-fed rats in both experiments. The inclusion of fat mass in this model is of interest as this was not changed by minocycline treatment, and fat mass measurements in CafVeh and CafMino rats overlap substantially. However, one reason for the strength of fat mass as a predictor of NPR performance may be its links with metabolic measures (plasma insulin and triglyceride concentrations) and hippocampal pro-inflammatory gene expression, which are known to be associated with diet-induced cognitive impairment and were impacted by minocycline treatment (Fig. 4j). It is noteworthy that *Desulfovibrio_*OTU31 had better predictive value for NPR than fat mass.

*Desulfovibrio*_OTU31 was consistently associated with NPR performance across independent cohorts of rats, and was putatively identified as a strain of *Desulfovibrio piger*, a Gram-negative, nonmotile bacillus. *Desulfovibrio* typically produce hydrogen sulfide and metabolize the sulfate moiety of sulfates, and their abundance positively correlates with increased adiposity and metabolic disease^53^. Another sulfate-reducing bacterium, *Desulfovibrio vulgaris* Hildenborough NCBI 8303, increased working memory errors in a radial arm maze and time spent finding the platform in a Morris water maze when administered to mice by gavage^54^. Contrastingly, intraperitoneal administration of sodium hydrosulfide has been shown to protect cognition in mice following surgery^55^ or in rats systemically injected with lipopolysaccharide (LPS)^56^, suggesting that some processes other than sulfate-reduction by *Desulfovibrio* species may negatively contribute to cognition.

Hippocampal gene expression of pro-inflammatory markers was elevated in vehicle-treated, cafeteria-fed (CafVeh) rats, in whom hippocampal *Il6* and *Il1B* expression were negatively associated with NPR performance. A recent study in humans found negative associations between serum IL-6 concentration and working memory^57^, and several preclinical rodent studies have shown that peripheral and central cytokine administration impair hippocampal-dependent forms of cognition^58,59^. Of particular note, we found that several pro-inflammatory genes were also elevated in minocycline-treated, chow-fed (CMino) rats, which may in part explain their poor performance on the NPR task; a result which warrants further investigation.

The small but consistent reduction in NPR performance observed in chow-fed rats exposed to minocycline is in agreement with previous work showing that large doses of broad-spectrum antibiotic cocktails impair cognition^60,61^, suggesting that the antibiotic activity of minocycline may underly this observation through changes in gut microbiome composition and function. Impaired cognition with minocycline treatment alone has not been reported previously in rats. To our knowledge, no studies have investigated cognition during chronic minocycline administration alone, but an acute intraperitoneal dose of minocycline exacerbated injury-associated cognitive impairment in a model of repetitive traumatic brain injury in neonatal rats ^62^.

Inflammatory gene expression in retroperitoneal WAT was not affected by minocycline treatment, consistent with the failure of minocycline to prevent the induction of WAT inflammation in a rodent model of acute pancreatitis^63^. However, *Tlr4* gene expression was significantly enriched in cafeteria-fed rats treated with minocycline only. TLR4 is an innate receptor for LPS^64^ and its expression is increased by both LPS and free fatty acid exposure^65^. The unique increase in this receptor’s expression in the WAT of cafeteria-fed rats treated with minocycline is most likely due to increases in circulating damage-associated molecular patterns following increased bacterial death, as minocycline targets both Gram-negative and Gram-positive bacteria. CafMino rats exhibited reduced bacterial species richness relative to all other groups at 2 weeks, providing evidence for a summative effect of cafeteria diet and minocycline treatment on bacterial species death.

Daily low minocycline treatment consistently reduced plasma triglyceride and insulin concentrations overall. Minocycline administration to rats with alloxan-induced diabetes reduced plasma triglyceride and blood glucose levels^66^ and lowering plasma triglyceride concentration in obese mice with gemfibrozil improved performance in the Morris water maze^67^. While these data suggest that reducing plasma triglyceride levels may improve cognition independent of other factors, it should be noted that plasma triglycerides in cafeteria-fed rats treated with minocycline remained statistically higher than both chow-fed groups. Further studies are required to determine whether such subtle reductions in plasma triglyceride concentrations as observed in the present study can improve cognition.

In terms of the translatability of these finding, minocycline is currently under investigation as adjunctive therapy for Alzheimer’s disease^68^, schizophrenia^69^ and multiple sclerosis^70^ with mixed results. While some studies report superiority over placebo, for instance in a metanalysis of adjunctive minocycline for schizophrenia^71^, others report little or no therapeutic benefit as well as issues of tolerability of a higher 400 mg dose^68^. Future work determining whether minocycline provides the same cognitive benefits to people with obesity as observed in these rodent studies, as well as investigations into long-term cognitive rescue, are warranted.

In summary, we show that oral minocycline treatment both prevents and improves spatial recognition memory impairments in cafeteria-fed rats while reducing hippocampal pro-inflammatory gene expression and disrupting fecal microbiome composition. Predictive modeling identified *Desulfovibrio*_OTU31 and fat mass as key factors for predicting short-term recognition memory in the context of cafeteria diet and minocycline exposure in rats.

## Supplementary information

Supplementary Figures and Tables

## Data Availability

The sequence data are available in the European Nucleotide Archive under accession number PRJEB34488. The study is currently set on private and will be released upon acceptance. All other data will be made available upon from the corresponding author on reasonable request.
