# Supplementary material for: Minocycline-induced microbiome alterations predict cafeteria diet-induced spatial recognition memory impairments in rats
Source: Transl Psychiatry. 2020 Mar 13;10:92. doi: 10.1038/s41398-020-0774-1 (PMC7069973; doi:10.1038/s41398-020-0774-1)
Supplement: Supplementary file 1 — Supplementary Figures and Tables [file 41398_2020_774_MOESM1_ESM.docx]

**Supplementary Figures and Tables**

**Supplementary Table 1.** Taq assay probe information

| **Gene name (abbreviation)** | **Unique assay identifier** |
| --- | --- |
| *Hippocampus* |  |
| Allograft inflammatory factor 1 (*Aif1*) | Rn00574125_g1 |
| Brain-derived neurotrophic factor (*Bdnf*) | Rn02531967_s1 |
| Claudin-5 (*Cldn5*) | Rn01753146_s1 |
| Glial fibrillary acidic protein (*Gfap*) | Rn01253033_m1 |
| Glucose transporter 1 (*Glut1*) | Rn01417099_m1 |
| Inhibitor of nuclear factor kappa B kinase subunit beta (*Ikbkb*) | Rn00584379_m1 |
| Occludin (*Ocln*) | Rn00580064_m1 |
| Synapsin 1 (*Syn1*) | Rn00569468_m1 |
| Tight junction protein 1 (*Tjp1*) | Rn02116071_s1 |
| Tropomyosin receptor kinase B (*Trkb*) | Rn01441749_m1 |
| Tyrosine 3-monooxygenase/tryptophan 5-monooxygenase activation protein zeta (*Ywhaz*) | Rn00755072_m1 |
| *Hippocampus and white adipose tissue* |  |
| Hypoxanthine phosephoribosyltransferase 1 (*Hprt1*) | Rn01527840_m1 |
| Interleukin-1 beta (*Il1B*) | Rn00580432_m1 |
| Interleukin-6 (*Il6*) | Rn01410330_m1 |
| Toll-like receptor 4 (*Tlr4*) | Rn99999017_m1 |
| Tumour necrosis factor alpha (*Tnf*) | Rn99999017_m1 |
| *White adipose tissue* |  |
| Cluster of differentiation 36 (*Cd36*) | Rn02115479_g1 |
| Glyceraldehyde 3-phosphate dehydrogenase (*Gapdh*) | Rn01749022_g1 |
| Interleukin-10 (*Il10*) | Rn00563409_m1 |
| Uncoupling protein 1 (Ucp1) | Rn00562126_m1 |


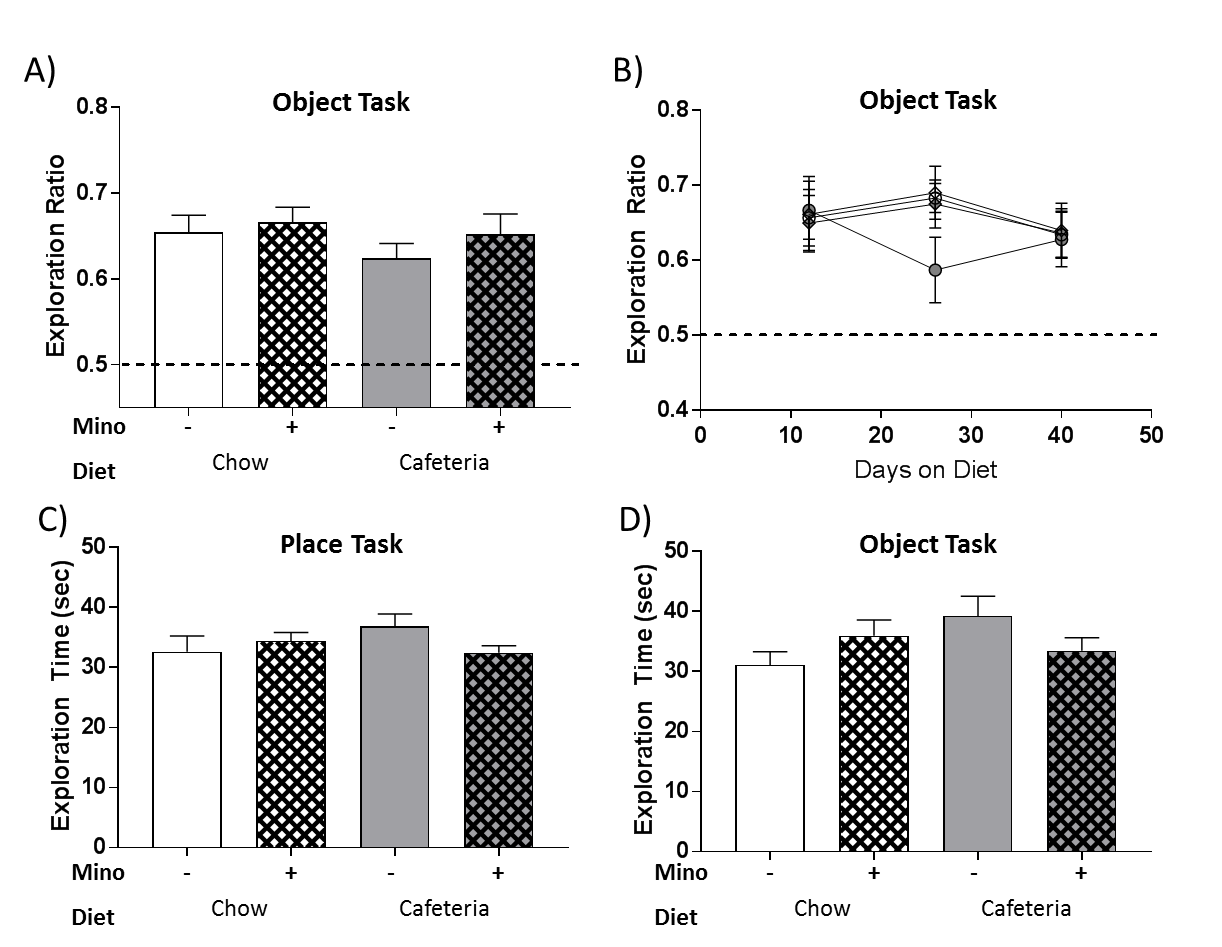


**Supplementary Figure 1.** Novel object recognition over time and exploration times.

(A) Average novel object task performance and (B) novel object performance over the study as exploration ratios. Average exploration times for (C) novel place tasks and (D) novel object tasks; results expressed as mean ± SEM; n=10-12.

**Supplementary Table 2.** Metabolic and gene expression variables correlated with place task performance

|  |  | Chow vs Cafeteria Diet | |
| --- | --- | --- | --- |
| Metabolic Variable | Overall | Vehicle | Minocycline |
| Fat mass | -0.291 (0.047) | -0.779 (<0.001) | 0.404 (0.051) |
| Lean mass | -0.47 (0.001) | -0.649 (0.001) | 0.025 (0.909) |
| Terminal body weight | -0.438 (0.002) | -0.749 (<0.001) | 0.329 (0.116) |
| Girth | -0.39 (0.007) | -0.76 (<0.001) | 0.374 (0.074) |
| Heart mass | -0.373 (0.01) | -0.605 (0.002) | 0.205 (0.338) |
| Total fat pad mass | -0.35 (0.016) | -0.799 (<0.001) | 0.348 (0.096) |
| Plasma insulin | -0.419 (0.004) | -0.589 (0.004) | 0.173 (0.430) |
| Plasma leptin | -0.395 (0.007) | -0.853 (<0.001) | 0.359 (0.092) |
| Plasma triglycerides | 0.093 (0.541) | 0.176 (0.423) | -0.032 (0.884) |
| Plasma high-density lipoprotein | 0.201 (0.181) | 0.327 (0.127) | 0.084 (0.705) |
| Retroperitoneal *Ucp1* | 0.399 (0.007) | 0.355 (0.105) | 0.511 (0.013) |
| Hippocampal *Il1b* | -0.197 (0.194) | -0.534 (0.013) | 0.123 (0.566) |
| Hippocampal *Il6* | -0.205 (0.176) | -0.454 (0.039) | 0.036 (0.866) |
| Hippocampal *Bdnf* | 0.189 (0.226) | 0.421 (0.064) | -0.100 (0.651) |

Pearson correlations between place task performance, and metabolic variables and hippocampal gene expression. Data expressed as Pearson correlation (p-value); Overall: N=48; all other columns: N=24.

**Supplementary Table 3.** Distance-based linear modelling to determine contributions of behavioral and metabolic measures to the variance observed in microbiota composition at the OTU level

| **4A – Marginal Model** | | | | | |
| --- | --- | --- | --- | --- | --- |
| Variable | SS(trace) | Pseudo-F | p-value | R^2^ |  |
| Diet | 20364 | 12.662 | 0.001 | 0.23595 |  |
| Drug | 9030.8 | 4.7915 | 0.001 | 0.10464 |  |
| Fat mass | 18817 | 11.432 | 0.001 | 0.21803 |  |
| Girth | 15754 | 9.1552 | 0.001 | 0.18254 |  |
| Liver mass | 13288 | 7.4615 | 0.001 | 0.15397 |  |
| Heart mass | 13647 | 7.7006 | 0.001 | 0.15812 |  |
| Naso-anal length | 5846.6 | 2.9793 | 0.007 | 0.067743 |  |
| Lean mass | 5527.4 | 2.8055 | 0.011 | 0.064044 |  |
| Plasma insulin | 8739.5 | 4.6195 | 0.001 | 0.10126 |  |
| Plasma leptin | 19219 | 11.746 | 0.001 | 0.22268 |  |
| Plasma triglycerides | 9054.4 | 4.8055 | 0.001 | 0.10491 |  |
| Retroperitoneal *Il6* | 6260.7 | 3.2068 | 0.002 | 0.072541 |  |
| Retroperitoneal *Tlr4* | 5404.6 | 2.739 | 0.013 | 0.062621 |  |
| Retroperitoneal *Ucp1* | 2848.9 | 1.3996 | 0.128 | 0.033009 |  |
| Hippocampal *Syn1* | 6606.1 | 3.3984 | 0.001 | 0.076543 |  |
| Hippocampal *Il1b* | 6563.4 | 3.3746 | 0.002 | 0.076048 |  |
| Hippocampal *Bdnf* | 7026.7 | 3.6339 | 0.002 | 0.081416 |  |
| Hippocampal *Glut1* | 3197.3 | 1.5773 | 0.092 | 0.037046 |  |
| Hippocampal *Tlr4* | 3028.8 | 1.4912 | 0.122 | 0.035094 |  |
| Hippocampal *Il6* | 2592 | 1.2694 | 0.198 | 0.030032 |  |
| Hippocampal *Cln5* | 2219 | 1.0819 | 0.296 | 0.02571 |  |
| Average place task performance | 5838.3 | 2.9747 | 0.006 | 0.067647 |  |
| Average object task performance | 4571.4 | 2.2931 | 0.023 | 0.052968 |  |

| **4B – Final Sequential Model** | | | | | |
| --- | --- | --- | --- | --- | --- |
| Variable | SS(trace) | Pseudo-F | p-value | R^2^ | Cumulative variance explained |
| Diet | 20364 | 12.662 | 0.001 | 0.236 | 0.236 |
| Drug | 8816.8 | 6.1737 | 0.001 | 0.102 | 0.338 |
| Fat mass | 3019.7 | 2.1767 | 0.009 | 0.035 | 0.373 |
| Plasma insulin | 2241.4 | 1.6423 | 0.019 | 0.026 | 0.399 |
| Plasma triglycerides | 2383.3 | 1.7821 | 0.007 | 0.028 | 0.427 |
| Hippocampal *Il6* | 1974.7 | 1.4965 | 0.032 | 0.029 | 0.450 |
| Average Place task performance | 2239.6 | 1.7316 | 0.007 | 0.026 | 0.476 |

Simultaneous and sequential distance-based linear modelling was used to investigate the unique and shared contributions of diet, minocycline treatment and variables of biological relevance on the variance observed in the microbiome composition (captured by the Bray-Curtis similarity matrix at the OTU level). Simultaneous distance-based linear modelling interrogates the unique contribution of each predictor variable to the variance explained in the Bray-Curtis similarity matrix and significant predictors identified by simultaneous regression are shown in the first table. Sequential multiple regression involves interrogating the conditional contribution of each variable in order of entry into the model (to determine whether variables contribute significantly to the variance explained in the presence of other variables); here, diet conditions were added before any metabolic predictors were considered and only the final model (only statistically significant covariates) is shown. Metabolic predictors included in the sequential regression were selected based on their predictive value, while trying to eliminate variables with high covariance; N=42-46.

**Supplementary Table 4.** Differentially expressed OTUs with minocycline treatment in cafeteria-fed rats following 6 weeks diet exposure

|  | DeSeq2 | | LefSe | |
| --- | --- | --- | --- | --- |
| OTU | log2FC | FDR | LDA Score | Enriched Group |
| *Prevotella*_OTU2 | -1.53 | 0.0490 | 4.13 | Minocycline |
| *Bacteroides*_OTU3 | 2.58 | <0.0001 | 4.14 | Vehicle |
| *Porphyromonadaceae unclassified*_OTU4 | 6.61 | <0.0001 | 3.78 | Vehicle |
| *Lactobacillus*_OTU5 | 4.60 | <0.0001 | 4.17 | Vehicle |
| *Bacteroides*_OTU9 | -1.24 | 0.0114 | 3.84 | Minocycline |
| *Blautia*_OTU12 | 2.21 | 0.0284 | 4.10 | Vehicle |
| *Porphyromonadaceae unclassified*_OTU16 | -3.39 | <0.0001 | 4.24 | Minocycline |
| *Lachnospiraceae unclassified*_OTU26 | -5.94 | <0.0001 | 3.89 | Minocycline |
| *Fusicatenibacter*_OTU30 | 8.01 | <0.0001 | 4.15 | Vehicle |
| *Desulfovibrio*_OTU31 | 7.98 | <0.0001 | 4.16 | Vehicle |
| *Parabacteroides*_OTU34 | -1.61 | 0.0010 | 3.76 | Minocycline |
| *Parasutterella*_OTU36 | 9.10 | <0.0001 | 4.07 | Vehicle |
| *Lachnospiraceae unclassified*_OTU39 | 3.94 | 0.0351 | 3.58 | Vehicle |
| *Lactobacillus*_OTU47 | 5.41 | <0.0001 | 3.71 | Vehicle |
| *Lactobacillus*_OTU53 | 7.11 | <0.0001 | 3.85 | Vehicle |
| *Sporobacter*_OTU57 | -3.59 | 0.0038 | 3.42 | Minocycline |
| *Lactobacillus*_OTU58 | 2.64 | 0.0001 | 3.51 | Vehicle |
| *Porphyromonadaceae unclassified*_OTU60 | -4.38 | <0.0001 | 3.57 | Minocycline |
| *Akkermansia*_OTU62 | 7.38 | <0.0001 | 3.78 | Vehicle |
| *Pseudoflavonifractor*_OTU65 | -2.89 | 0.0013 | 3.54 | Minocycline |
| *Porphyromonadaceae unclassified*_OTU67 | -2.18 | 0.0004 | 3.81 | Minocycline |
| *Ruminococcus*_OTU75 | 8.36 | <0.0001 | 3.92 | Vehicle |
| *Clostridium XlVa*_OTU84 | 2.59 | 0.0052 | 3.70 | Vehicle |
| *Porphyromonadaceae unclassified*_OTU85 | -2.58 | 0.0451 | 3.47 | Minocycline |
| *Ruminococcaceae unclassified*_OTU88 | -1.75 | 0.0243 | 3.61 | Minocycline |
| *Clostridiales unclassified*_OTU95 | -3.13 | <0.0001 | 3.73 | Minocycline |
| *Ruminococcaceae unclassified*_OTU97 | -4.61 | <0.0001 | 3.74 | Minocycline |
| *Porphyromonadaceae unclassified*_OTU116 | -6.00 | 0.0000 | 3.90 | Minocycline |
| *Lachnospiraceae unclassified*_OTU139 | 3.61 | <0.0001 | 3.72 | Vehicle |
| *Blautia*_OTU140 | 2.30 | 0.0201 | 3.62 | Vehicle |
| *Lachnospiraceae unclassified*_OTU146 | -3.22 | 0.0212 | 3.38 | Minocycline |
| *Clostridium XlVa*_OTU189 | -3.97 | <0.0001 | 3.66 | Minocycline |

OTUs of interest among the top 200 identified to differ significantly in abundance by DESeq2 (adjusted p<0.05) and LEfSe (LDA score>2, p<0.05) at the 6-week time point; n=12.

**Supplementary Table 5.** Energy intake, anthropometric measures at tissue collection and plasma measures for Experiment 2

| Measure | Chow Diet | | Cafeteria Diet | | P-Values | | |  |
| --- | --- | --- | --- | --- | --- | --- | --- | --- |
|  | Vehicle | Minocycline | Vehicle | Minocycline | Diet | Mino | Interaction |  |
| Total Energy and Macronutrient Intake (average kJ/rat) | | | | | | | | |
| Total energy intake | 19313 ± 79 | 20004 ± 37 | 68121 ± 276 | 68050 ± 348 | <0.001 | - | - |  |
| Total protein intake | 12538 ± 51 | 12985 ± 24 | 13602 ± 58 | 13785 ± 65 | 0.033 | - | - |  |
| Total carbohydrate intake | 12536 ± 51 | 12985 ± 24 | 38943 ± 180 | 38775 ± 229 | <0.001 | - | - |  |
| Total fat intake | 2598 ± 11 | 2691 ± 5 | 20691 ± 76 | 21373 ± 124 | <0.001 | - | - |  |
| Anthropometric Measures | | | | | | | |  |
| Terminal body weight (g) | 526.67 ± 20.10 | 535.18 ± 11.91 | 693.99 ± 24.53 ^a,b^ | 671.61 ± 16.65 ^a,b^ | <0.001 | - | - |  |
| Naso-anal Length (cm) | 25.7 ± 0.3 | 26.4 ± 0.2 | 27.0 ± 0.3 ^a^ | 26.8 ± 0.2^a^ | 0.003 | - | 0.068 |  |
| Girth (cm) | 19.3 ± 0.3 | 18.9 ± 0.2 | 21.6 ± 0.5 ^a,b^ | 21.5 ± 0.4 ^a,b^ | <0.001 | - | - |  |
| Tibia length (cm) | 4.34 ± 0.04 | 4.36 ± 0.03 | 4.46 ± 0.06 | 4.36 ± 0.04 | <0.001 | - | - |  |
| Body composition at 8 weeks | | | | | | | | |
| Fat mass (%) | 10.6 ± 1.1 | 9.3 ± 0.9 | 23.0 ± 1.5 | 21.4 ± 1.5 | <0.001 | - | - |  |
| Lean mass (g) | 423.4 ± 13.4 | 440.8 ± 8.4 | 470.1 ± 13.2 | 473.7 ± 11.2 | 0.001 | - | - |  |
| Organ Weights | | | | | | | |  |
| Liver Weight (g) | 17.70 ± 0.94 | 17.75 ± 0.74 | 24.69 ± 1.36^a,b^ | 27.17 ± 1.27^a,b^ | <0.001 | - | - |  |
| Heart Weight (g) | 0.99 ± 0.04 | 1.00 ± 0.02 | 1.15 ± 0.03^a,b^ | 1.17 ± 0.02^a,b^ | <0.001 | - | - |  |
| Fat Pad Weights | | | | | | | |  |
| Retroperitoneal (g) | 6.63 ± 1.19 | 5.44 ± 0.66 | 21.84 ± 2.21^a,b^ | 20.09 ± 1.68^a,b^ | <0.001 | - | - |  |
| Gonadal (g) | 6.17 ± 0.77 | 5.94 ± 0.87 | 20.37 ± 1.34 ^a,b^ | 18.57 ± 1.46 ^a,b^ | <0.001 | - | - |  |
| Total (g) | 12.80 ± 1.90 | 11.38 ± 1.50 | 42.21 ± 3.34 ^a,b^ | 38.66 ± 3.05 ^a,b^ | <0.001 | - | - |  |

Data expressed as mean ± SEM; n=4 cages for energy intake measures; n=10-12 for other measures. Data was analyzed using two-way ANOVA, followed by post-hoc multiple comparisons with a Tukey HSD correction.

^a^p<0.05 relative to CVeh, ^b^p<0.05 relative to CMino, ^c^p<0.05 relative to CafVeh
